# Social Functioning and Autistic Behaviors in Youth Following Acquired Brain Injury

**DOI:** 10.3390/children9111648

**Published:** 2022-10-28

**Authors:** Rachel K. Greene, Natalia Rich-Wimmer, Cydni N. Williams, Trevor A. Hall

**Affiliations:** 1 Department of Pediatrics, Division of Pediatric Psychology, Oregon Health and Science University, Portland, OR 97239, USA; 2 Department of Pediatrics, Division of Critical Care, Oregon Health and Science University, Portland, OR 97239, USA; 3 Pediatric Critical Care and Neurotrauma Recovery Program, Oregon Health and Science University, Portland, OR 97239, USA

**Keywords:** acquired brain injury, pediatric, social functioning, autism

## Abstract

Children and adolescents who survive the pediatric intensive care unit (PICU) with an acquired brain injury (ABI) often demonstrate a variety of physical, cognitive, emotional/behavioral, and social sequelae termed post-intensive care syndrome (PICS). Social communication and interaction challenges have also been observed clinically, and there is growing literature documenting these occurrences in youth following ABI. The extent of these social changes varies among patients, and a subset of patients go on to exhibit social and behavioral profiles closely resembling those of autistic youth. We reviewed empirical research regarding social functioning in youth following ABI, as well as the overlap between individuals with ABI and autistic youth, published from January 2009 to August 2022 on PubMed and Scopus databases. Clinical case examples from a well-established post-PICU follow-up program are also provided to exemplify the complexity of this phenomenon.

## 1. Introduction

Acquired brain injury (ABI) is a prevalent cause of mortality and morbidity in young children. ABI is the result of a variety of primary etiologies including, but not limited to, head trauma, stroke, seizure, infection, and cardiac arrest that require specialized critical care services to optimize outcomes. In fact, ABI from a primary neurologic diagnosis accounts for >20% of all pediatric intensive care unit (PICU) admissions and more than 60,000 hospital admissions annually [1,2,3,4,5]. ABI can also occur secondary to PICU-acquired morbidities such as delirium, neuroactive medication exposure, and prolonged immobilization, which are often associated with a number of medical, cognitive, behavioral, and academic sequelae for pediatric patients [3,6,7,8]. Cognitive outcomes have been well-studied in youth with acquired brain injuries such as sudden cardiac arrest, stroke, and traumatic brain injury [9,10]. Research findings show that children with these aforementioned presentations commonly experience ongoing performance deficits in the cognitive domains of executive functioning, attention regulation, processing speed, memory, and the utilization of adaptive skills that impacts learning at school and overall neurodevelopmental trajectories [11,12,13]. Further, while much of the cognitive impact studied in neurologic populations is related to severity, it is important to note that even patients with *mild* brain injury are at risk for chronic cognitive deficits, and that age and developmental stage at time of injury likely affect response to intervention and an individual patient’s trajectory of recovery.

One often-overlooked aspect of recovery in youth who experience ABI is social functioning, despite the importance of social competence in academic pursuits, relational success, community functioning, and general well-being [14,15,16]. While social impacts have been explored to some degree in adults with ABI [17,18], there are relatively fewer investigations regarding social functioning following ABI in pediatric samples. It is important to recognize that social skills and competency are acquired through a developmental process. Childhood and adolescence are developmental periods associated with gradually increasing social demands and specific social pressures (e.g., peer influence, avoiding social rejection; [19], along with substantial changes in brain structure that directly mediate social behavior. Therefore, children and adolescents who experience ABI may be at heightened risk, relative to adults, for disruption of their social development trajectory from both an environmental and biological perspective.

While certain brain regions have been identified as playing primary roles in specific social functions (i.e., prefrontal cortex, anterior cingulate cortex, temporo-parietal junction, posterior temporal sulcus, insula, and amygdala; [20,21], those brain regions, along with others, act within a complex network to process and implement socially motivated behaviors in everyday life [22]. Therefore, insults to those brain regions and those neural circuits at any point could lead to observable social changes. Furthermore, social functioning is influenced by a number of factors including, but not limited to, reduced social opportunities, executive functioning, communication abilities, and emotion regulation [23,24,25]. Consequently, disruptions to those functions or diminished opportunity for social interaction in general may also have indirect effects on social behavior broadly.

Finally, a subset of youth who have experienced ABI go on to exhibit a number of social and behavioral features that are commonly observed in autistic individuals^†^. Autism spectrum disorder (ASD) is a behaviorally defined, heterogeneous neurodevelopmental condition characterized by differences in social communication and interaction as well as restricted and repetitive behaviors. It is hypothesized that there are many factors (e.g., biological, environmental, genetic) that contribute to and result in the behavioral profile we call autism. Therefore, changes to neural functioning resulting from ABI may present an autistic behavioral phenotype in some individuals. The aims of the current manuscript are to:

(1) Outline the social challenges experienced by children and adolescents following ABI (updating the systematic review conducted by [26]),
(2) Discuss factors that contribute to social outcomes in youth who experience ABI, and
(3) Describe the overlap in clinical profiles of some individuals with ABI and autistic individuals and present several clinical case examples that exemplify changes in social functioning following ABI.

## 2. Methods

### 2.1. Review

We conducted a narrative review and selected the PubMed (Medline) and Scopus electronic databases for searching. This specific review was not registered. Two filters were selected in both search databases: (1) studies published between January 2012 to August 2022 and (2) English as language of publication. The first author was responsible for reviewing which studies met inclusion and exclusion criteria.

**Figure 1:**
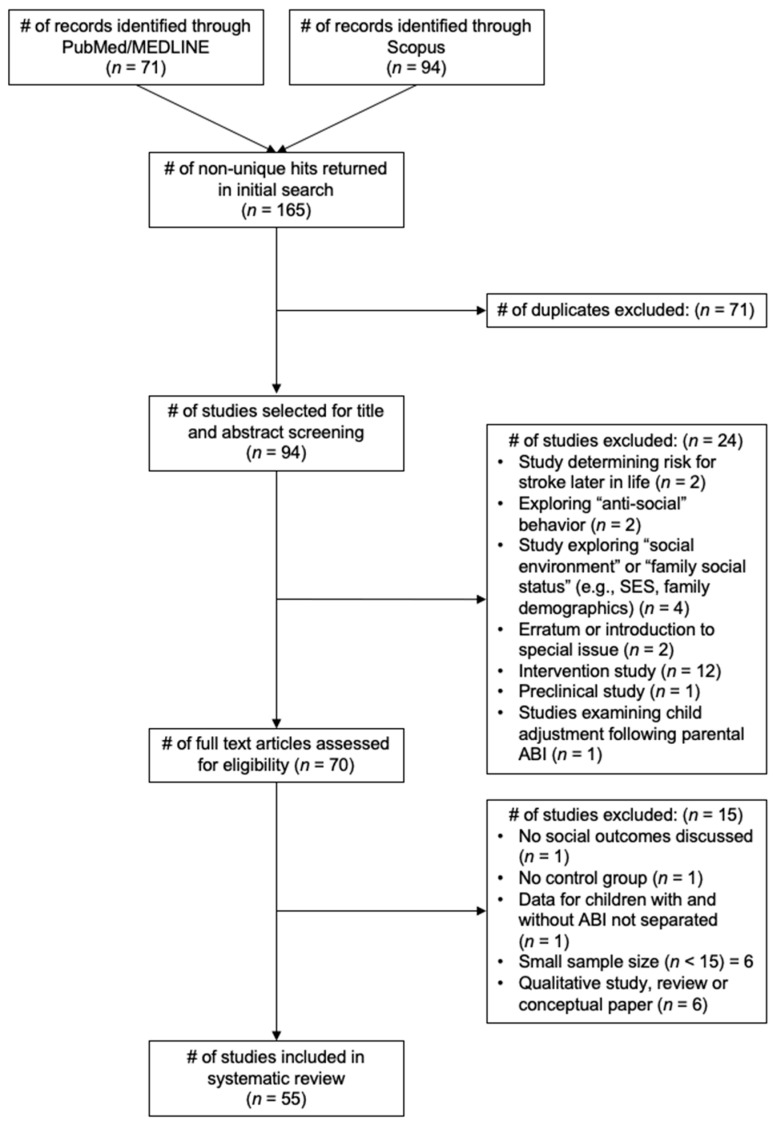
PRISMA Flow Diagram. *Note:* ABI = acquired brain injury; SES = socioeconomic status, # = number.

The following terms/combinations were used to search article titles: (“brain injury” OR “acquired brain injury” OR “traumatic brain injury” OR “tbi” OR “stroke” OR “meningitis” OR “encephalitis” OR “cardiac arrest” OR “anoxi*” OR “hypoxi*”) AND (“social” OR “theory of mind” OR “social cognition” OR “social behav*r” OR “social adjustment” OR “social interaction” OR “pragmatic language” OR “emotion* recognition” OR “fac* recognition” OR “social communication”) AND (“infant” OR “child*” OR “teen*” OR “adolescent*” OR “pediatric” OR “youth”). This search yielded 94 unique findings (see Figure 1). Empirical studies with *n* ≥ 15 were included in the final review when they directly reported social outcomes as primary research findings and included a control or comparison group. Reviews and qualitative studies were examined but excluded from the following literature review. Several studies were excluded, as their content was not directly relevant to the current topic. For example, some studies referred to “anti-social” behavior (e.g., aggression, violence, rule breaking, etc.) or “family social status” rather than examining individual social functioning more broadly. Intervention studies were also excluded, as this was outside of the scope of the current review. With the current search terms and exclusionary criteria, a total of 55 studies are included in the literature review below. Notably, no studies were found regarding the social abilities of youth who have experienced meningitis, cardiac arrest, hypoxic or anoxic brain injuries, or encephalitis.

### 2.2. Case Exemplars

Clinical case examples for this review come from observations made during clinical visits from a well-established post-PICU follow-up program at a regional academic medical institution in the United States. The information contained within has been modified as to avoid the possibility of identification. Nonetheless, the core clinical features presented remain true to the cases and are intended to bridge the research contained within this review to real-world clinical practice.

## 3. Social Outcomes in Youth Following ABI

Of our identified studies, all were conducted since [26] initially completed their systematic review on the social functioning of children and adolescents following TBI that spanned studies from 1989 to 2011. More recently, a systematic review and meta-analysis surrounding social cognitive outcomes in a pediatric TBI sample was conducted by On and colleagues [27]. The following results aim to build on those works and expand those findings to include acquired brain injury more broadly, as well as other social constructs beyond social cognition alone. Although these various social constructs are presented below as unique and defined variables, they are thought to be linked and interconnected, playing off of one another in everyday social exchanges [28].

### 3.1. Social Adjustment and Participation

Ashton [29] defined social adjustment as “subjective social satisfaction and social acceptance within a group” and noted, “… both are influenced by the individual’s behavior and social context” (p. 176). Understandably, the degree of social adjustment often has direct impacts on the quality of that individual’s social relationships and participation. Anderson and colleagues [30] described a four-factor model of social adjustment following TBI in children. First, social brain networks may be disrupted by an injury; second, medical factors (e.g., mobility difficulties, speech changes, seizures) limit access to social interactions; third, the child’s temperament dictates how they adjust to stress and various environmental changes following their injury; and, finally, these factors are impacted by family stress, parenting styles, family resources, and other environmental factors.

A review by Gauvin-Lepage, Lapierre, and Bissonnette [31] described the number of ways in which ABIs negatively impact the frequency and quality of social participation in children and adolescents. In alignment with Anderson’s model, the review describes how injury-specific (e.g., increased severity), internal (e.g., decreased tolerance of social rejection, lower cognitive functioning), and external factors (e.g., poorer parental mental health, lower socioeconomic status) negatively impact youth social participation following ABI. Further, a number of studies have reported poorer social adaptation and decreased social participation in youth following stroke [32,33,34], with challenges sometimes persisting in the absence of cognitive and/or behavioral difficulties. Similarly, 40% of parents of children who have survived bacterial meningitis and septicemia reported significant social and/or behavioral sequalae following the illness, and many of the families felt the social and behavioral aspects of recovery were under-recognized and underserved [35].

Children with TBIs have also demonstrated poor social adjustment and reduced social participation [36,37,38], and this may be particularly pronounced for patients with more severe injuries [39]. Poorer social adjustment has been associated with increased externalizing behaviors, whereas decreased social participation was linked to internalizing behaviors. Social participation and adjustment were also shown to be impacted by social communication difficulties, indicating a possible area for intervention. Social participation, relative to other social constructs, may be particularly vulnerable in the months immediately following injury given medically mandated activity restrictions and then later improve (particularly in more mild-to-moderate cases) while other social difficulties (e.g., social cognitive, social communication) persist [40]. Notably, children with severe TBIs describe themselves as being significantly less socially rejected/victimized than their peers rate them as being, indicating that difficulties with social awareness may accompany broader social challenges [41]. Ratings of children with mild-to-moderate TBI did not differ significantly from their peers. Peer reports have also proven to be a better mediator of the relationships between executive functioning, social behavior, and peer acceptance in children with TBI when compared to teacher reports. While we often think of how these injuries impact peer-to-peer relationships, one study also showed observational differences in parent–child interactions following TBI [42].

### 3.2. Social Cognition and Social Information Processing

Social cognition broadly encompasses our ability to perceive and interpret environmental social cues such as language and nonverbal communication (e.g., facial expression, eye gaze, vocal intonation, and gestures; [24,43]. Social cognitive skills include the ability to infer the social–emotional states or beliefs of others (i.e., Theory of Mind (ToM), emotion recognition, and social problem solving). ToM can be further divided into affective (i.e., inferring the emotional states of others), cognitive (i.e., inferring the beliefs and perspectives of others), and conative (i.e., concerned with influencing another’s thoughts or feelings) abilities. Social cognitive impairments have frequently been documented in youth following TBI [44], and to a lesser extent in children with ABI, such as stroke or neurological infection; however, this has been observed clinically. A recent systematic review and meta-analysis regarding social cognitive functioning in pediatric TBI patients [27] found that many youths who experience TBI are vulnerable to declines in higher-order social cognitive abilities such as pragmatic language, social problem solving, and ToM. Social information processing also involves leveraging broader executive functioning skills that are often impacted by ABI.

ToM impairments are the most well-documented social cognitive changes following ABI [44,45,46] and have been observed in individuals irrespective of injury severity and from preschool to late adolescence [47]. Tousignant et al. [48] revealed adolescents with moderate-to-severe TBI reported and demonstrated impaired perspective taking and ToM abilities compared to controls. These findings remained at least marginally significant when accounting for nonsocial higher-order cognitive abilities. Dennis et al. [49] reported that only children with severe TBI exhibited cognitive ToM deficits relative to orthopedic injury controls, whereas both children with mild-to-moderate and severe TBI showed affective ToM and conative ToM challenges. Alternatively, another study solely observed ToM difficulties (cognitive, affective, and conative) in children with mild-to-moderate TBI, whereas the same effects were not observed in children with severe TBI [50]. Affective ToM deficits in children with TBI have been associated with peer-rated social rejection-victimization [28]. Children with severe TBI have also shown relative differences in their social problem-solving strategies relative to youth with mild-to-moderate TBI and controls [51]. Processing speed difficulties have also been associated with poorer social participation in children with TBIs [39]. While characterizing ToM impairments is challenging, it is clear that executive functioning skills and ToM appear to mediate the relationship between TBI and social adjustment [52]. Similarly, children with arterial ischemic stroke (AIS) demonstrate difficulties with cognitive, affective, and conative ToM [53,54]. Adding to the complexity, a number of studies have identified broader executive functioning skills as playing an important role in peer acceptance and social competence for youth with a history of ABI [55], and peer-reported sociability and prosociality, as well as teacher-reported prosociality, positively mediate the relationship between executive functioning and peer acceptance, whereas social ostracism/victimization negatively mediate the relationship.

Expanding from ToM to emotional perception is also conceptually difficult. Although studies involving youth with TBI show little evidence of impaired emotion perception or recognition relative to controls [56,57,58], basic emotion perception difficulties have been observed in individuals who sustained more moderate-to-severe TBIs in childhood [59,60], suggesting the severity of injury may contribute specifically to emotion-recognition abilities following TBI. Ryan and colleagues [60] also found that emotion-recognition abilities were positively correlated with posterior corpus collosum volume. Difficulties with interpreting more nuanced nonverbal cues that conflict with semantic messaging have been observed in young adults who sustained childhood TBIs, irrespective of severity [60]. Children with AIS also exhibit difficulties with basic facial emotion processing [54]. Reading more subtle nonverbal signals is key to one’s ability to effectively utilize social-pragmatic communication. This skill, as described above, is a more developmentally advanced task than basic emotion recognition; therefore, it follows that those abilities might be preferentially impacted while emotion recognition skills are spared in patients with more mild-to-moderate injuries. Further, social-pragmatic language is also often disrupted in children following TBI, and has been shown to mediate the relationship between early-childhood TBI and social and conceptual adaptive functioning in middle childhood relative to children with orthopedic injuries alone [61]. Middle childhood TBI has also been associated with sustained pragmatic language difficulties 24-months postinjury and was associated with increased externalizing behaviors [62].

### 3.3. Neural Correlates of Social Functioning Following ABI

Along with technological advances in neuroimaging have come an emerging understanding of the neural correlates of social changes following ABI in children and adolescents. Although little-to-no neuroimaging research has been conducted regarding social neural underpinning in children with broader ABI (e.g., stroke, neural infection, etc.), there have been several related investigations of canonical social brain regions, as well as global brain volume, following pediatric TBI. For example, children and adolescents who had experienced severe TBI demonstrated reduced volume of brain regions within the “Social Brain Network” (SBN; [63]. The volumetrically diminished regions included the superior temporal sulcus, fusiform gyrus, temporal pole, medial prefrontal cortex, frontal pole, orbitofrontal cortex, temporo-parietal junction, cingulate, and amygdala. The insulas of individuals with TBI were also marginally smaller (*p* = 0.062). The severe TBI group also demonstrated poorer ToM performance relative to mild TBI patients and the control. ToM abilities were associated with more frequent behavioral problems and abnormal neural morphology. Similarly, Yeates and colleagues [28] found that reduced global brain volume was positively correlated with ToM abilities in children with and without TBI. Findings of widespread volumetric reductions and specific reductions of regions within the mentalizing network and their association with poorer ToM abilities following pediatric TBI have been corroborated [64,65]. Specifically, reductions in regions within the cerebro-cerebellar mentalizing network were associated with poorer cognitive ToM, whereas reduced volume of regions within the salience network and mirror neuron/empathy network were associated with greater difficulty with affective and conative ToM. Given that many children with more severe ABIs experience broad neural atrophy [66,67], this could represent one mechanism by which social functioning declines postinjury.

Ryan et al. [68] later conducted a study examining neural diffusion in children with mild TBI by using diffusion tensor imaging. Relative to the control group, TBI youth showed higher mean diffusivity, axial diffusivity, and radial diffusivity, with differences seen most prominently in the splenium of the corpus callosum, sagittal stratum, dorsal cingulum, uncinated fasciculus, and middle and superior cerebellar peduncles. Their findings also associated ToM impairments with diffuse neuropathology and parietal lobe lesions following pediatric TBI [69]. Reductions in thickness and surface area of the corpus callosum have also been associated with social pragmatic communication difficulties in youth following TBI [69,70]. Children with TBI showed poorer affective ToM and pragmatic language at 24-months postinjury, and those outcomes were related to previously observed alterations in diffusivity of the dorsal cingulum and middle cerebellar peduncle. The authors hypothesized that dysfunction within frontal-limbic and cerebro-cerebellar connectivity may contribute to social cognitive challenges observed in youth following TBI. Similarly, increased resting-state connectivity has been observed between SBN regions, including prefrontal-fusiform and fusiform-superior frontal connections [71]. These neural connectivity findings were not significantly associated with parent-reported behavioral or social functioning. However, these findings are corroborated by an observed association with increased cerebral blood flow to prefrontal brain regions and simultaneous increased activation of posterior regions while completing a social cognition task in adolescents following moderate-to-severe TBI [72]. Right frontal pole cortical thickness was also associated with parent-reported social problems in children with a history of TBI [73], and that relationship was moderated by cognitive processing abilities (i.e., working memory and processing speed; [74]. Together, these findings repeatedly illustrate that networks typically recruited to process social information may be disrupted following ABI. More specifically, most findings showed that connections and activations are amplified and disorganized in nature.

## 4. Factors That Influence Social Outcomes and Resilience Following ABI

Clearly, the literature documents a strong association between ABI and social dysfunction for many children and adolescents. Therefore, for clinicians and researchers alike, a logical next question remains: how do we predict who might go on to develop social challenges as a result of their injury and, therefore, require additional support and interventions? Studies show that there are several early predictors of later social disruption in the months and years following ABI. Work in this area thus far has shown that long-term trajectories post-ABI are determined via a complex interplay between injury-specific/timing factors, preinjury functioning, and environmental context [37,39,40,75,76].

Severe injuries are often, but not always, associated with more significant declines in social functioning [75,77]. However, this does not mean that individuals who sustain mild or moderate injuries show no social sequalae. In fact, patients without cognitive decline or behavioral changes also report changes in social abilities. Alternatively, individuals with more severe injuries and recovery courses go on to return to baseline social functioning, indicating injury severity is not necessarily deterministic one way or another. These trajectories and their complexity were exemplified by Anderson and colleagues [75], who identified five distinct recovery trajectories in children 2-years post-TBI. The trajectories were described as Impaired (7%), Slow Recovery (15%), Intact (42%), Early Recovery (7%), and Resilient (29%). The impaired and slow recovery groups showed declines in social functioning within the first 12 months. While the Slow Recovery group returned to preinjury social functioning, the Impaired group’s impairments persisted through the next year. Interestingly, the groups did not significantly differ from one another regarding injury severity, Glasgow Coma Scale (GCS), or affected brain region(s). The findings are variable regarding the impact of age at injury on social outcomes. It is hypothesized that the interaction between age at injury and social outcomes is not necessarily linear in nature. Rather, due to increased neural plasticity in early development serving as a protective factor, a critical period model in which the specific timing of an injury variably impacts outcomes [27,78] is most appropriate. For example, Crowe and colleagues [79] identified TBIs occurring in middle childhood (7–9 years old), as opposed to infancy, preschool, and late childhood, as being associated with the poorest cognitive performance. Similarly, social and behavioral functioning appears to be most disrupted following TBIs in middle childhood [62]. Some studies have shown greater socialization difficulties for children who experienced childhood-onset stroke (≥29 days old at the time of stroke) versus neonatal onset stroke [80]. A similar pattern was reported by Anderson and colleagues [32] for children following stroke. Contrastingly, others have suggested more significant social impacts for children with TBIs and ABIs occurring in earlier developmental periods [36,39,81], suggesting the type of injury in combination with the developmental timepoint at which it occurs is meaningful for social outcomes.

As illustrated in the preceding paragraphs, injury-specific and timing factors are important when considering the potential for social impact related to brain injury. Just as important are individualized factors related to a given child’s preinjury/illness developmental status and ability to regulate emotions and behavior. In a sample of children with mild-to-severe TBI, parent-reported behavioral regulation abilities 6 months postinjury were predictive of poorer social skills and social adjustment 12 months postinjury [38], suggesting regulatory abilities may be important predictors of later social dysfunction. In fact, Kaldoja and Kolk [82] found that parents of young boys who sustained TBIs rated them as having more self-regulation difficulties than peers who did not have TBIs prior to injury on the Ages and Stages Questionnaire (ASQ) completed within a routine pediatric visit. Therefore, the relationship between self-regulation, TBI, and social dysfunction may be multidirectional in nature. This is consistent with the literature that shows a higher incidence of TBI in individuals with Attention-Deficit/Hyperactivity Disorder (ADHD), a condition characterized by behavioral disinhibition [83]. There has also been increased awareness of the role of sex and gender on recovery from ABI [84]. Specifically, girls who experience TBI report sharper decreases in self-confidence and social initiation following their injury relative to their male peers, whereas boys are more likely to show decreases in social participation [31]. The work in this area is limited but warrants future research.

While the five trajectories identified by Anderson and colleagues [75] did not differ from one another in terms of age, sex, injury severity, GCS, or brain region involvement, they were differentiated by environmental factors such as preinjury, two-year family dysfunction, and two-year perceived family burden. Specifically, family environmental indexes were most impacted within the Impaired group. Interestingly, the Intact group had some environmental risk factors (e.g., borderline parent mental health at two years, elevated family function scores at baseline at two years); however, the fact that those scores and the level of familial distress did not appear to change over time as a result of the injury suggested limited family dysfunction as a result of the TBI itself, which may be an important factor. An earlier study using the same measure found no impact of family functioning on TBI social outcomes [40]; therefore, it may be that family functioning acts in an indirect way to impact recovery broadly. While various studies have hypothesized that lower socioeconomic status (SES) may negatively impact social recovery following pediatric ABI, most studies found no evidence of such influence [37,40,75]. Familial factors that may affect social functioning following ABI include family dysfunction, parenting style, parental education, and parent mental health [33,40,85]. For example, parental mental health predicted more internalizing and social problems, as well as lower social participation, in a group of pediatric stroke patients [33]. Additionally, low levels of maternal nurturance have been shown to moderate the relationship between injury status (i.e., TBI vs. TDC) and peer rejection [86]. These findings broadly suggest that interventions supporting the family as a whole may be appropriate and effective after a child experiences an ABI.

Overall, Anderson’s trajectories and the extant literature highlight that social resilience is broadly associated with a combination of intact family and parent functioning, better preinjury adaptive abilities, and postinjury cognition and social participation. Alternatively, vulnerability in the social domain was related to poorer pre- and postinjury adaptive abilities, greater behavioral concerns, and poorer pre- and postinjury parent mental health and family function. The findings remain unclear as to how factors such as age at injury and gender specifically impact social outcomes, and more work in these areas is needed.

## 5. Overlap between ABI and ASD

Autism is a neurodevelopmental disorder characterized by core differences in social communication and interaction, as well as restricted and repetitive behaviors and sensory differences [87]. As previously mentioned, children with ABI may show reduced social participation, poorer social adjustment, increased social ostracism/victimization, and social communication difficulties. Therefore, it is not surprising that, clinically, a subset of patients with ABI show similar social profiles to autistic individuals. In fact, the construct of social cognition, which is a noted challenge for many children with ABI, was first conceived and developed by examining psychiatric conditions such as ASD and schizophrenia, where social cognitive challenges are quite pronounced [17]. ASD research has also yielded robust literature on the neural underpinnings of the core social features of the condition, though there is still much to explore. Similar to the ABI literature, ASD researchers have examined volumetric, functional activation, and resting-state functional connectivity patterns within SBN regions. Broadly, a pattern of reduced resting-state functional connectivity and reduced activation in regions in response to social stimuli has been observed in autistic individuals [88]. Atypical SBN structural features have also been observed, including reduced volume of superior temporal sulcus, fusiform gyrus, and amygdala [89].

Given the interconnectivity among the SBN regions and their vulnerability to neurological injury and illness, it is understandable how the presentations of individuals with ABI who experience significant social changes following their injuries might mimic those of autistic individuals. Nonetheless, social communication and interaction differences alone are not sufficient for a diagnosis of ASD. Individuals must also present with two of four restricted and repetitive behaviors (RRBs): (1) repetitive behaviors, motor movements, or speech; (2) restricted, unusual, and/or highly intense topics of interest; (3) insistence on sameness or routine; and (4) sensory difference (e.g., hypo- or hypersensitivity). For individuals with ABI, these repetitive behaviors may present themselves as new-onset stereotyped motor mannerisms [90], strong topics of interest or obsessions [91], perseverative verbal patterns [92], and sensory sensitivities (most often to noise and light; [93]. Notably, many of the studies demonstrating increased RRBs following ABI involved injuries to frontal brain regions, suggesting injuries to this area may preferentially confer risk for such sequelae. However, the circuits involved in RRBs are thought to be broader, including cerebral cortex, basal ganglia, and cerebellum [91]. Additionally, alternative explanations for emergent RRB symptoms should be explored. For example, working memory deficits have been shown to be related to perseverative speech patterns in individuals with TBI [92], and anxiety, which is common for individuals after a traumatic medical event, may result in an increased preference for routine and consistency.

Beyond sharing some of the defining features of ASD, individuals with ABI also exhibit a number of peripheral but common cognitive (e.g., learning and executive functioning difficulties), medical (e.g., seizures, gastrointestinal symptoms), and behavioral symptoms (e.g., behavioral dysregulation). Singh and colleagues [94] provided an extensive overview of these commonalities as well as the possible shared mechanisms between the two conditions. Certainly, the shared neurophysiological differences in global and social brain network regions point to a likely explanation for the phenotypic overlap.

Of course, while there are similarities between ABI and ASD, there are differences as well. One major distinction between the two is the timing at which neural substrates are impacted. ASD is considered a life-long condition, present from birth, whereas ABI can occur after a period of typical development. Certainly, some ABIs occur in utero or in infancy, and, in that way, may be more closely aligned with ASD presentations in terms of the downstream developmental impacts of living with social, cognitive, and/or behavioral differences from or since shortly after birth. Additionally, many ABI patients may exhibit social changes without the requisite RRBs for an ASD diagnosis. Their presentations may, therefore, align more closely with a diagnosis of Social Pragmatic Communication Disorder in those cases. A small study (i.e., N = 20) did find that parents of children with ABI and ASD endorsed a similar number of social differences on the Social Communication Questionnaire (SCQ; [95]), but patients with ABI presented with fewer RRB-specific symptoms [96]. These preliminarily findings suggest that RRBs may be what differentiates ABI from ASD when significant social concerns are present. Altogether, very little research has investigated RRBs in this population; therefore, the prevalence of such behaviors is relatively unknown outside of isolated studies with smaller samples and case reports. Additional research is needed.

## 6. Abstracted Case Exemplars

As part of the Pediatric Critical Care and Neurotrauma Recovery Program (PCCNRP) at Doernbecher Children’s Hospital, youth cared for in the PICU with a neurologic injury or illness attend an initial postdischarge follow-up clinic at four to six weeks postdischarge from the hospital. At that appointment, all youth receive a physical and neurological examination from a pediatric critical care physician. Likewise, all youth participate in a brief neuropsychological assessment with a neuropsychologist. These evaluations occur in an integrated manner with findings shared in real-time and feedback provided to families with both providers present. The goal for the initial PCCNRP appointment is to check up on how the youth and their family are doing since they left the hospital so that an individualized treatment plan can be developed with the intention of optimizing recovery. Roughly half the patients seen are referred to the PCCNRP long-term recovery clinic, which provides traditional comprehensive neuropsychological evaluations for youth 9-to-12 months postdischarge from the hospital, with the continued goal of informing a treatment plan to optimize recovery. The PCCNRP referrals, follow-up patterns, and program details have been previously described [9,10]. The following abstracted case examples come from patients that have participated in both the initial and long-term PCCNRP recovery clinics.

### 6.1. Meningitis—Subdural Empyema

An 11-year-old, right-handed male with pre-existing diagnoses of attention deficit/hyperactivity disorder (ADHD), generalized anxiety disorder, panic attacks, and sensory integration dysfunction was hospitalized following the development of a progressive right-sided spastic hemiplegia and dysarthria. Initial brain imaging (CT scan) revealed a left frontal subdural empyema with an incidental finding of a right frontal dermoid cyst. The child underwent a left frontotemporal parietal craniotomy with evacuation of the convexity and frontal subdural empyema. Follow-up MRIs redemonstrated the right inferior frontal lobe dermoid cyst as stable without residual fluid collections. Developmentally, the child met developmental motor milestones within age-appropriate time limits. However, developmental language milestones were relatively delayed (i.e., delayed speech and difficulty with speech articulation); however, no interventions were prescribed. Difficulties regarding intelligibility were resolved by 3 years of age. Academically, the patient received in-home instruction. His mother reported satisfactory academic performance without formal academic accommodations or interventions prior to his hospitalization. During his comprehensive neuropsychological evaluation, the child displayed difficulties with executive functioning skills (i.e., attention, planning and organizing, cognitive flexibility, working memory, and abstract reasoning), processing speed, expressive language, fine motor coordination, and spelling. At the time he was diagnosed with ADHD, mild neurocognitive disorder due to his acquired brain injury, specific learning disorder, and an unspecified anxiety disorder. In contrast, during a second comprehensive neuropsychological re-evaluation two-years postdischarge, the patient’s mother reported ongoing and escalating concerns with low frustration tolerance, social communication difficulties, behavioral rigidity (i.e., insisting on daily wearing of a specific t-shirt), and restricted interests (i.e., always carrying certain objects with him). Observationally, the child presented as highly apprehensive of separating from his mother. He also demonstrated inconsistent eye contact and nonverbal gestures, with poor reciprocal conversation skills. Difficulty with sustained attention, hyperactivity, and self-monitoring requiring consistent use of structured breaks was also noted. Results of the evaluation highlighted significant difficulties with dynamic reciprocal social interactions, rigidity to rituals and routines, and sensitivity to sensory input, which were attributed to an additional diagnosis of ASD. The diagnostic team conceptualized the case, as the child had some pre-existing risk factors which may have been amplified over time due to network disruptions across the prefrontal cortex. The deficits became clearer over time as he aged and the executive/social demands of his life increased.

### 6.2. Brain Hemorrhage—Brain Arteriovenous Malformation

A 9-year-old, left-handed male with history of premature birth and a pre-existing diagnosis of ADHD and developmental delay presented to the emergency department after complaining of a headache, vomiting, and becoming unresponsive. Initial diagnostic imaging (MRI) revealed a left cerebellar intraparenchymal hemorrhagic mass with mass effect and left-to-right midline shift in the posterior fossa, with further imaging revealing arteriovenous malformation (AVM). After discharge, updated brain imaging (MRI) showed reduced size of the known posterior fossa hemorrhage with decreased compression on the fourth ventricle. Two months postdischarge, the child completed a suboccipital craniotomy for resection of the AVM; his postoperative angiogram indicated total resection and he was weaned off of Keppra prophylaxis. Developmentally, postpartum complications included a month-long admission in the neonatal intensive care unit for feeding and respiratory distress. Early childhood intervention services were utilized to support development. With regard to academics, the patient historically performed similarly to peers his age. During his follow-up comprehensive neuropsychological evaluation, his parents noted increased difficulty with frustration tolerance and negative thinking patterns which exacerbated social and emotional challenges (e.g., social initiation, atypical style of social communication, rigid thought patterns and fixated areas of interests, difficulties with transitions, as well as sensory-avoiding and -seeking behaviors). Observationally, the patient demonstrated difficulty with social reciprocity, perseveration on specific topics of interest, echolalia, and inattention, requiring tangible behavioral reinforcement techniques. Results of the evaluation indicated weaknesses in executive function (i.e., self-monitoring, set shifting, attention, and abstract reasoning) which were consistent with his premorbid diagnosis of ADHD. However, he also met the diagnostic criteria for a diagnosis of ASD. Given the nature of the child’s brain injury, it was considered possible that the AVM in the cerebellum was present and insidiously disruptive prior to being recognized. Given the location of the AVM, cognitive, language, motor, sensory, and emotional functions associated with conditioned reflex responses, mental imagery, anticipatory planning, aspects of attention, affective behavior, visual spatial organization, and the control of sensory data acquisition were likely impacted and contributed to clinical features commonly associated with ASD.

### 6.3. Traumatic Brain Injury

A typically developing 9-year-old, right-handed female experienced a mild-complex TBI. Initial diagnostic imaging (CT scan) revealed a right-sided temporo-parietal depressed skull fracture and an epidural hematoma requiring craniotomy for evacuation. A postoperative Quick Brain-MRI revealed a right posterior frontal/anterior parietal lobe contusion. Within a month of being discharged home, the child presented to a local emergency department for headache and fever, with a reassuring exam and Quick Brain-MRI that showed evolving encephalomalacia in the right parietal lobe. Prior to her hospitalization, the child met her developmental milestones on time, but she required informal small group instruction in school for reading and writing. Immediately following her TBI, teachers noted increased difficulty with gross motor coordination, forgetfulness, and difficulty with efficiently completing tasks. During her follow-up comprehensive neuropsychological evaluation, her parents noted concerns related to emotional lability (e.g., low frustration tolerance, easily becoming overwhelmed, and crying), difficulties with executive function (i.e., attention and concentration, planning and organizing, and cognitive flexibility), fine motor dexterity, processing speed, and memory recall. Parents also noted changes in personality (e.g., appearing shyer and with reduced self-esteem) following her TBI (risk factors for social withdrawal). Observationally, the patient demonstrated relative discomfort when being interviewed regarding post-traumatic symptoms. However, she exhibited age-appropriate eye contact and nonverbal gestures as well as good social and emotional reciprocity. Results of the evaluation indicated weaknesses in executive function (i.e., self-monitoring, set shifting, and attention), visual-spatial abilities, visual organization skills, and visual working memory which were consistent with the focal nature of her injury to the right posterior frontal/anterior parietal lobe. This child’s mild preinjury neurodevelopmental vulnerabilities (i.e., learning concerns), in concert with the location of her contusion and the resultant evolving encephalomalacia, led to some consideration about characterizing her newer cognitive and emotional challenges via an ASD phenotype. This concern was also raised by professionals within her school environment. In the end, it was clinically determined that her cognitive and emotional sequelae were best characterized by a diagnosis of mild neurocognitive disorder due to traumatic injury with behavioral disturbance. This case demonstrates how some ASD symptomology might be observed in similar injury presentations but might not always be the best diagnostic description.

## 7. Future Directions

It is important to note that a majority of the studies identified in the current literature search involved patients with TBI specifically and, to a lesser degree, pediatric stroke. Very little works of literature have been published regarding other ABIs such as neurological infections, anoxic or hypoxic brain injury, cardiac arrest, or encephalitis in pediatric populations. This suggests more research is needed to examine the social impact of these pediatric brain injuries specifically. Additionally, the neural and behavioral overlap between ABI and ASD has been relatively unexplored. In addition to the social communication and interaction differences seen in both, there are other commonalities (e.g., perseverative thoughts and speech, sensory sensitivities) as well that warrant further investigation. This avenue of research could help guide clinicians regarding not only diagnosis but appropriate interventions and long-term support for children and families.

## 8. Conclusions and Considerations

Decreased social participation, poor social adjustment, and social cognitive deficits (including impairment in ToM) have been observed in a subset of youth with ABI, sometimes in the absence of cognitive and/or behavioral sequalae, suggesting that more mild presentations require ongoing monitoring and support for social functioning. Further, given the occurrence of social concerns following ABI and neuroimaging studies documenting neurophysiological differences in key social brain network regions, it makes sense that the behavioral profiles of some children with ABI mimic those of autistic children.

Regarding whether or not it is appropriate to provide an ASD diagnosis following ABI remains up for debate. Some may argue that the etiology and developmental course of disability in ABI is so dissimilar from that of the life-long condition of ASD, particularly for those with ABI who experience late childhood- or adolescent-onset injuries, that they could not be considered under the same label. Others subscribe to the notion that ASD is, under our current diagnostic standards, a behaviorally defined disorder that in many ways disregards etiology whether it be due to injury, genetic abnormality, or idiopathic occurrence. A perspective in line with an RDoC framework [97] might de-emphasize labels altogether, identify areas of need, and recommend appropriate treatment that suits the presenting concerns. This might be a particularly salient approach because, as discussed in this review, social outcomes are influenced by a complex interplay between preinjury internal and external variables, as well as injury-specific factors (e.g., type and severity of injury). Additionally, the existing literature suggests, if indicated, appropriate intervention should be provided not only to the individual but to the family. In the end, the field must examine what benefit a label of ASD would provide to children with ABI in the context of our current medical system and for whom it may be appropriate. Would this label change the course of treatment or access to applicable, needed services? A handful of studies have described employing social skills interventions, such as those that are commonly geared toward autistic youth, for children following TBI [98,99]. Others have adopted applied behavior analysis (ABA) and behaviorally based interventions to help pediatric TBI patients adjust to home life after their injury [100]. Future research should explore these various perspectives and intervention approaches.

What is clear is that, like ASD, ABI is characterized by significant neuropsychological and medical heterogeneity, such that changes in social, behavioral, cognitive, adaptive, and emotional functioning vary highly across individuals. Because of this, individualized and tailored assessment, monitoring, and intervention is required to ensure children and adolescents with ABI and their families are wholly supported from the time of initial injury through long-term recovery.
